# Low Dose Ribavirin for Treatment of Hepatitis C Virus Infected Thalassemia Major Patients; New Indications for Combination Therapy

**DOI:** 10.5812/hepatmon.6592

**Published:** 2012-06-30

**Authors:** Seyed Vahid Tabatabaei, Seyed Moayed Alavian, Maryam Keshvari, Bita Behnava, Seyyed Mohammad Miri, Pegah Karimi Elizee, Farhad Zamani, Sedigheh Amini Kafiabad, Ahmad Gharehbaghian, Bashir Hajibeigy, Kamran Bagheri Lankarani

**Affiliations:** 1 Baqiyatallah Research Center for Gastroenterology and Liver Disease, Baqiyatallah University of Medical Sciences, Tehran, IR Iran; 2 Iranian Blood Transfusion Organization Research Centre (IBTO), Tehran, IR Iran; 3 Liver Disease Research Center, Iran University of Medical Sciences, Tehran, IR Iran; 4 Shiraz University of Medical Sciences, Shiraz, IR Iran

**Keywords:** Beta-Thalassemia, Hepacivirus, Ribavirin, Peginterferon Alfa-2a

## Abstract

**Background:**

Treatment guidelines contraindicate ribavirin for treatment of hepatitis C virus (HCV) infection in thalassemia major patients. Nevertheless, the current evidence suggests that ribavirin might be tolerated by these patients.

**Objectives:**

Despite this evidence, low dose ribavirin combination therapy has not been compared with peginterferon monotherapy in these patients so far.

**Patients and Methods:**

Two hundred eighty thalassemia patients with detectable HCV-RNA PCR (≥ 50 IU/mL) and liver histology consistent with chronic HCV infection were self-assigned to receive peginterferon alfa-2a (n = 81) monotherapy or its combination therapy with ribavirin, 600-800 mg QD, according to hemoglobin levels (n = 199). Treatment experienced patients were eligible for this study.

**Results:**

Sustained virological response (SVR) was significantly higher in patients who received ribavirin (51 % vs. 38 % P = 0.02). In multivariate regression, OR of ribavirin for prediction of SVR was 2.2 (95 % CI 1.24-3.91). The SVR was significantly higher in the ribavirin group in subgroups of patients with more than 24 years of age, elevated ALT, ferritin < 2006 ng/mL, previous treatment failure, genotype 1, positive history of splenectomy, fibrosis score of 0-4 HAI and viral load < 600,000 IU/mL. Treatment discontinuations due to the safety concerns were comparable between the treatment groups (6.5 and 8 %). Furthermore, transfusion intervals were almost halved in patients who received low dose ribavirin.

**Conclusions:**

According to the present study, adult thalassemia patients with HCV infection can be treated successfully with low dose ribavirin. Hence, we strongly advise combination therapy in thalassemia patients with aforementioned clinical characteristics. Moreover, ribavirin does not seem to be beneficial in thalassemia patients below 18 years of age.

## 1. Background

Chronic hepatitis C (CHC) is a major cause of morbidity and mortality in thalassemia major patients, particularly in those who received their first transfusion before the introduction of HCV donor screening program [1]. Since sustained virological response (SVR) to anti-HCV therapy avoids fibrosis progression, decreases the risk of HCC, and improves patients’ survival, it is a crucial option in management of these patients [2][3][4][5]. In patients without hemoglobinopathies, current guidelines strongly recommend combination therapy of ribavirin and either peginterferon alfa-2a or 2b. This combination therapy can yield a SVR rate of more than 50 % in genotype 1 and 70-80 % of SVR in genotype 2/3 infected patients [6][7][8][9][10]. In contrast to patients without inherent hemoglobinopathy, ribavirin, that is one of the major determinants of the SVR, can induce life-threatening anemia in thalassemia major patients, and thus, is generally considered contraindicated in these patients [11][12]. In addition to contraindication of ribavirin, high iron content of serum and liver with its probable role in conferring patients’ interferon resistance and its synergistic effect on progression of liver fibrosis is another major confounder in treatment of thalassemia major patients [13][14][15][16][17][18][19]. Previously in a meta-analysis of literature, we showed that peginterferon monotherapy can induce a SVR rate of 28 percent in thalassemia major patients, while combination therapy of peginterferon and ribavirin (low dose therapy, accompanying by tight monitoring of patients) elicits a SVR rate of 44 percent [20]. The small sample size and lack of control group in included studies were two major downsides of our meta-analysis [21][22][23][24][25][26]. Indeed, this study has been designed to address this issue and to reach a more precise estimation of ribavirin tolerability and the effect of its low dose administration on improving SVR in HCV infected thalassemia major patients.

## 2. Objectives

In current study peginterferon alfa-2a plus low dose ribavirin combination therapy has been compared with peginterferon alfa-2a monotherapy in a head to head open-labeled clinical trial of 280 patients. This huge number of patients could let us determine major subgroups that benefit the most from low dose ribavirin.

## 3. Patients and Methods

### 3.1. Patient Selection

280 thalassemia major patients with quantifiable serum HCV RNA levels (> 50 IU/mL) and liver biopsy findings consistent with diagnosis of chronic HCV infection were enrolled in our study. our exclusion criteria were as follows: 1) hepatitis B virus or HIV co-infection, 2) decompensated liver disease, 3) hepatocellular carcinoma, 4) bone marrow or liver transplant, 5) creatinine clearance < 50 mL/min, 6) poorly controlled psychiatric disorder, 7) poorly controlled diabetes, 8) malignant neoplastic disease, 9) severe cardiac or chronic pulmonary disease, 10) active substance abuse, 11) immunologically mediated disease, or 12) retinopathy. Patients with previous treatment failure following conventional interferon with or without ribavirin therapy were still eligible.

### 3.2. Study Design

This study was designed as a single centre, prospective open-label controlled trial of peginterferon alfa-2a (Pegasys®) and ribavirin (COPEGUS®) combination therapy in HCV infected thalassemia major patients. Sponsors of this study and the academic principal investigators were jointly responsible for the study design and its protocol development. This study has been conducted at Iranian blood transfusion organization hepatitis clinic, and has been registered in www.clinicaltrials.gov (identifier: NCT00707850). Eligible subjects were self-assigned to two treatment groups. Patients in the group (A) received 180 μg of Pegasys® subcutaneously once a week in combination with oral ribavirin 600-800 mg per day according to patients’ hemoglobin level. Patients with a hemoglobin level of 8-10 g/dL received 600 mg ribavirin, whereas those with hemoglobin level of over 10 g/ dL received 800 mg Rbavirin. Genotype 1 and mixed infection were treated for a total duration of 48 weeks, while patients with genotype 2 or 3 were treated for 24 weeks. Subjects in group (B) received 180μg of Pegasys® subcutaneously once a week for duration of 48 weeks. As ribavirin is not approved for administration to patients with hemoglobinopathies, randomization and blinding of patients to treatment regimens were recognized unethical and were avoided. Therefore, all patients underwent consultation regarding the potential benefits and hazards of ribavirin in aggravation of their underlying disease and its likely significant effects on their anti-HCV treatment outcome. Consequently, 199 subjects accepted to receive ribavirin and were assigned to group (A) and 81 subjects that declined were assigned to group (B). Meanwhile, thalassemia major had been confirmed in all patients with hemoglobin electrophoresis or DNA testing earlier in their life. In addition, all subjects had been receiving regular blood transfusions at two to four week intervals along with regular therapy with defroxamine to maintain hemoglobin levels at 10–13 g/dL. Compliance with treatment was monitored using telephone consultation, questionnaires and returned vials for peginterferon alfa2a. The protocol was approved by the ministry of health appointed Protocol Review Committee as well as the Iranian Blood Transfusion organization and Baqiyatallah University of Medical Sciences Review Boards. All patients provided written informed consent. Informed consent was also obtained from parents of patients younger than the legal age (< 18 years).

### 3.3. Liver Histology

Patients underwent percutaneous liver biopsy by Menghini needles. Each biopsy specimen was evaluated according to the modified Knodell score grading and staging system by a single pathologist who was blind to patients’ clinical and laboratory data and treatment regimens. The Perls’ staining method with 0-4 score was applied to assess hepatic siderosis. 50 subjects with liver biopsy results of older than two years ago declined to perform a new liver biopsy. Thus, their data of liver histology were considered missing in the statistical analysis.

### 3.4. Assessment of Efficacy

Our primary efficacy end point was the sustained virological response, defined as undetectable HCV RNA after 24 weeks of untreated follow-up. Our secondary efficacy end points were early virological response (EVR) defined as negative HCV-RNA after 12 weeks of treatment and end of treatment response (ETR) which is negative HCV RNA at the end of treatment. According to the established guidelines, patients with an insufficient virological response at 12th week (a detectable HCV RNA level and a decrease of < 2 log_10_ IU/mL from the baseline level) and a detectable HCV RNA level at 24th week of treatment were considered to show treatment failure and were withdrawn from treatment. Analysis included data from all patients who received at least one dose of studied medications.

### 3.5. Assessment of Safety

Safety was assessed by monthly laboratory tests and evaluation of adverse events during treatment and follow-up period in group (B). on the other hand, patients’ hemoglobin level was checked every week in group (A), until the recognition of transfusion necessity and afterward, according to the pattern of identified hemoglobin drop. Adverse events were graded by the investigators as mild, moderate, severe, or life-threatening, according to a modified world health organization (WHO) grading system. Non–life-threatening adverse events were managed by reduction of the dose of peginterferon alfa-2a, or with appropriate medical treatments. The laboratory criteria for dose reduction of peginterferon alfa-2a or treatment discontinuation were as follows: 500-750 neutrophils/ mm^3^) and 30000-50000 platelets /mm^3^ for dose reduction and decline of neutrophil count to below 500/mm^3^ and platelet count to below 30000/mm^3^ for treatment discontinuation. Furthermore, the interval between transfusions was reduced if it was necessary to maintain the hemoglobin level between 10 and 13 g/dL. Whenever the patients developed an abnormal laboratory test, they were asked to return and to be retested at an interval of one or two weeks, in addition to dose adjustment or temporary treatment discontinuation. Afterwards, further modifications were introduced to treatment regimen or treatment was even discontinued according to the patients’ condition.

### 3.6. Virological Methods

HCV genotyping was carried out according to the method that has been described previously [27]. Viral load quantification was performed using the Cobas Amplicor HCV Monitor, v2.0 (Roche Diagnostics, Branchburg, NJ, USA) with a lowest detection level of 50 IU/mL HCV RNA.

### 3.7. Statistical Analysis

The current trial was designed to detect clinically meaningful differences of the rates of SVR and patients’ safety between two treatment regimens in general and in subset of patients. Statistics are summarized in tables for each of the two treatment regimens. Continuous variables are presented as mean values ± standard deviation (SD), while qualitative and discrete variables are presented as absolute and relative frequencies in the form of percentage. Chi-squared test was applied to assess associations between categorical variables. The comparisons between continuous and qualitative variables were performed by student’s t-test. Multivariable logistic-regression analyses involving treatment regimen, baseline characteristics and previous treatment history were performed to study SVR in all studied populations. A stepwise procedure was used to identify independent predictors of SVR with P = 0.05 as the threshold level for variables to be entered into and retained in the final model, and P = 0.1 as the threshold level for variables to be removed. All computations were carried out using SPSS version 16.

## 4. Results

### 4.1. Characteristics of Studied Patients

Totally, 280 patients were included in the present study, with their age ranging from 11 to 54 years (mean: 24.2 ±0.3 years). From them, 60 % were male. The mean body weight of patients was 51.5 ± 0.6 kg (ranging between 23 and 81 kg). Average of serum ALT values was 2.2 times the upper limit of the normal range. Mean baseline viral load was 774,000 IU/mL and 24 % had an HCV RNA level of > 1 million IU/mL. 71 % were infected with genotype 1. Liver necro inflammation was mild in 135 patients (59 %), while it was moderate and severe in 81(35 %) and 9(4%) patients respectively. From the other side, liver fibrosis was mild in 66 (29 %), moderate in 62 (30 %) and bridging in 81 (35 %) patients, whereas 21 patients (9 %) had cirrhosis. Baseline demographic characteristics were balanced between the two treatment groups (Table 1). However, patients in group (A) who received ribavirin had a significantly higher rate of previous treatment failure, serum ferritin and liver enzymes.

**Table 1:** Baseline Characteristics of Studied Patients a, b

	**Group A**	**Group B**	***P value***
Patients, No.	199	81	
Gender			0.1
Male, No.	123	42	
Female, No.	76	39	
Mean, %	62	60	
Mean Age , Mean ± SD	24 ± 5.5	25 ± 7.2	0.3
range	11-43	12-54	
BMI c, Mean ± SD	20.4 ± 0.2	20.2 ± 0.2	
range	14-28	15-25	
ALT c (U/L) , Mean ± SD	91 ± 56	79 ± 60	0.06
range	12-994	15-338	
Normal (< 40 U/L), No. (%)	35 (18)	24 (30)	0.02
Elevated, No. (%)	164 (82)	57 (70)	0.02
AST c (U/L) , Mean ± SD	77 ± 61	64 ± 41	0.08
range	17-638	13-206	
Normal (< 40 U/L), No. (%)	43 (22)	29 (36)	0.01
Elevated, No. (%)	156 (78)	62 (64)	0.01
Hb c, Mean ± SD	10 ± 1.5	9.5 ± 1.3	0.4
HCV c viral load d (IU/mL) , Mean ± SD	800000 ± 11000	799000 ± 88000	
range	2000-8000000	7540-4090000	
Log_10_ Serum HCV Viral Load (IU/mL) , Mean ± SD	5.5 ± 0.7	5.5 ± 0.6	0.5
> 6 (copy/ml), No. (%)	85 (43)	31 (38)	
Serum ferritin (ng/mL) , Mean ± SD	2130 ± 1777	1710 ± 1498	0.06
range	210-8132	300-6650	
History of Splenectomy, y, No.(%)			
Yes/No	135/64 (68)	49/32 (60)	0.2
HCV Type			
Genotype 1, No. (%)	119 (60)	41 (51)	0.1
Genotype 2, No. (%)	1 (0.5)	2 (2.5)	0.1
Genotype 3, No. (%)	65 (33)	33 (41)	0.1
Mixed infection, No. (%)	10 (5)	2 (2.5)	0.1
Untypable, No. (%)	4 (2)	3 (4)	0.1
Stage of liver fibrosis , Mean ± SD	3.2 ± 1.6	3.3 ± 1.4	0.5
0-2, No. (%)	50 (31)	16 (20)	0.3
3-4, No. (%)	69 (43)	37 (53)	0.3
5-6, No. (%)	41 (26)	17 (24)	0.3
Grade of liver inflammation , Mean ± SD	6.3 ± 0.2	6.3 ± 0.4	0.9
0-6, No. (%)	92 (59)	43 (63)	0.7
7-12, No. (%)	58 (37)	23 (34)	0.7
13-18, No. (%)	7 (4.5)	2 (3)	0.7
Stage of liver sidrosis , Mean ± SD	3 ± 1	2.9 ± 1	
0-2	37 (19)	16 (20)	0.2
3-4	118 (59)	34 (42)	0.2
Previous treatment	136 (68)	38 (47)	0.001
Naïve	61 (31)	43 (53)	0.0006
Standard IFN c	62 (31)	24 (30)	0.8
Standard IFN + RVB c	74 (37)	14 (17)	0.001

^a^ Except for liver enzymes and serum ferritin there were no significant differences among the two treatment groups with regard to baseline characteristics.

^b^ Percutaneous liver-biopsy specimens obtained before treatment were evaluated according to modified knodell score scaling system. The modified knodell scoring system classifies fibrosis according to a 6-point scale: 0, no fibrosis; 1, Fibrous expansion of some portal areas; 2, Fibrous expansion of most portal areas; 3, Fibrous expansion of most portal areas with occasional portal to portal bridging; 4, Fibrous expansion of portal areas with marked bridging of portal to portal as well as portal to central; 5, Marked bridging with occasional nodules; 6, Cirrhosis.

^c^ Abbreviations: ALT, denotes alanine aminotransferase; AST, aspartat aminotransferase; BMI, body mass index; Hb, hemoglobin; HCV, hepatitis C virus; IFN, interferon; RVB, ribavirin

^d^ Hepatitis C virus level was determined with the use of the amplicor assay version II (Roche), for which the lower limit of quantitation is 50 IU per milliliter.

### 4.2. Virologic Response Rates and Treatment Efficacy

**Figure 1:**
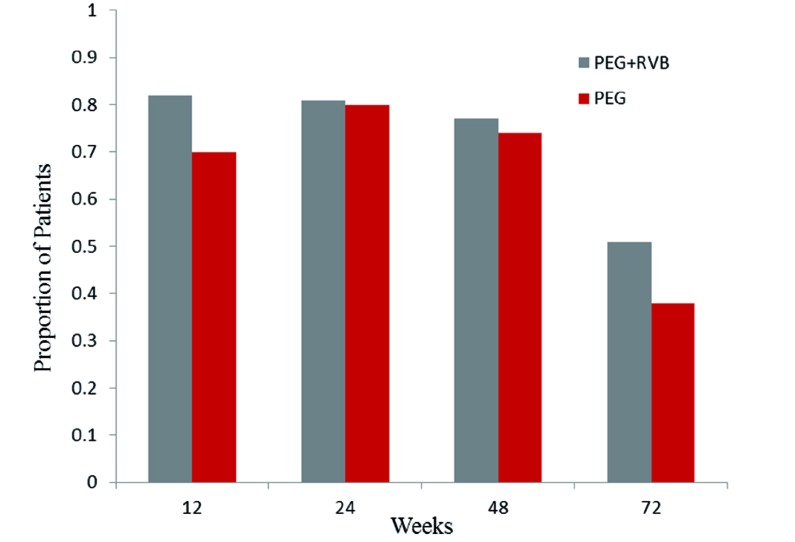
Virologic Response (HCV-RNA < 50 IU/Ml) Through Treatment and 24 Weeks Late

**Figure 2:**
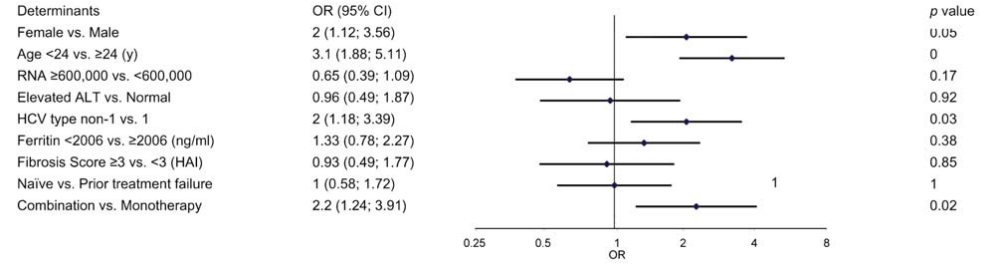
Results of Stepwise Multivariate Logistic Regression Including Baseline Characteristics to Predict a SVR in All Studied Population

**Figure 3:**
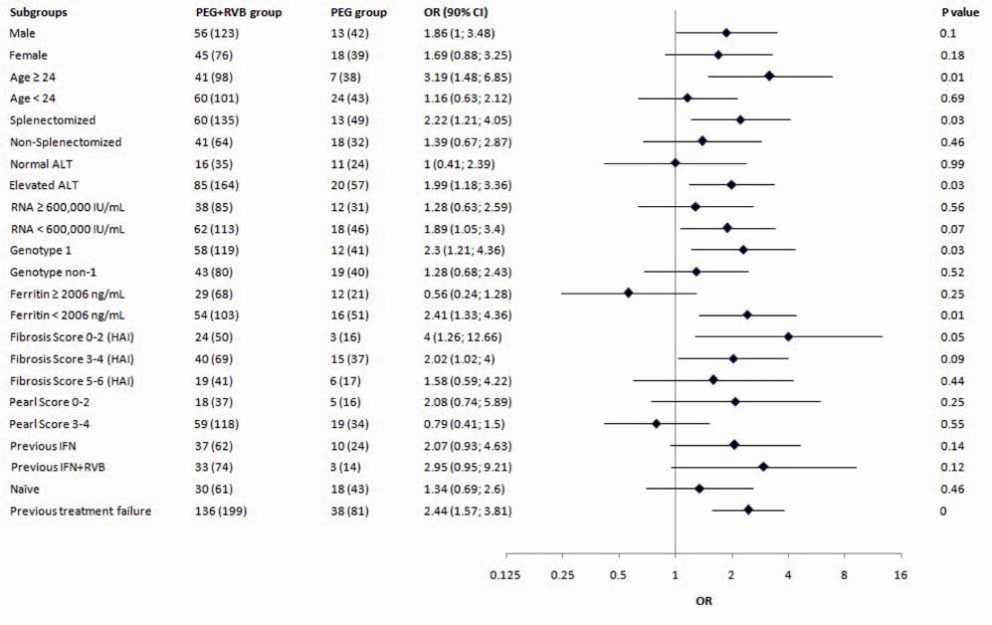
The Effect of Treatment Regimen on the Rate of SVR in Subgroups of Patients (The Chance of Type I Error Was Increased to 0.1 in Response to Low Statistical Power)

Treatment was administered through 24 or 48 weeks according to the HCV type and treatment regimen. Figure 1 shows the virological response which is defined as an undetectable serum hepatitis C virus (HCV) RNA level (< 50 IU/mL) during the study period in 199 patients with combination therapy and 81 patients who received monotherapy. Patients with missing HCV RNA measurements at the various time points were considered as not having a response. The primary end point of SVR was measured at 24 weeks after treatment cessation. The percentage of patients with SVR was 51 % (95 % CI, 44 to 58) for group (A) and 38 % (95 % CI, 27 to 48) for group (B). The estimated difference in SVR rates was 13 % (95 % CI 2-23). Response rates at the end of the treatment phase did not differ between groups (A) and (B). However, virological relapse rate was significantly higher in patients who received peginterferon alfa-2a monotherapy (35 % vs. 23 %). our primary analysis in a multiple logistic model controlled for all baseline characteristics revealed that the rate of SVR was significantly higher in patients who were given low dose ribavirin therapy [OR = 2.2 95 % CI 1.24-3.91]. In both groups, HCV RNA suppression (negative HCV RNA with qualitative PCR at treatment week 12) was strongly associated with a sustained virological response (P < 0.0001) and was also significantly higher in the group (A) (Figure 1). Stepwise multivariable logistic-regression analyses identified several baseline factors such as being treated with ribavirin, female gender, age < 24 years and HCV type as independent predictors of sustained virological response (Figure 2). The odds ratios of the SVR among patients who received combination therapy to patients who received monotherapy according to baseline characteristics are shown in Figure 3. A far more pronounced effect of low dose ribavirin was observed in patients with one of the following baseline characteristics: age ≥ 24, elevated ALT level, history of splenectomy, HCV genotype 1, viral load < 600,000 IU/mL, serum ferritin < 2006 ng/mL, liver modified-HAI fibrosis of 0 to 4 and previous treatment failure.

### 4.3. Drug Doses and Side Effects

**Figure 4:**
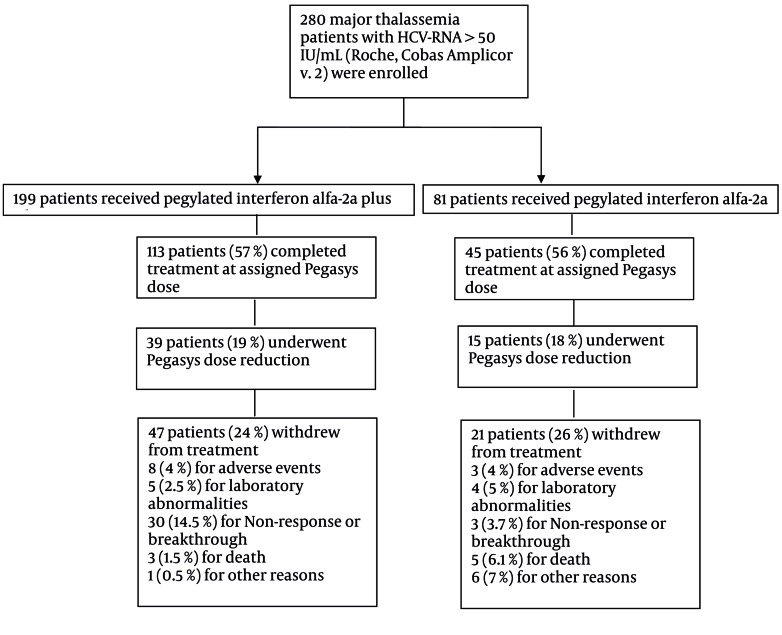
Enrollments and Follow-up of the Study Patients

**Figure 5:**
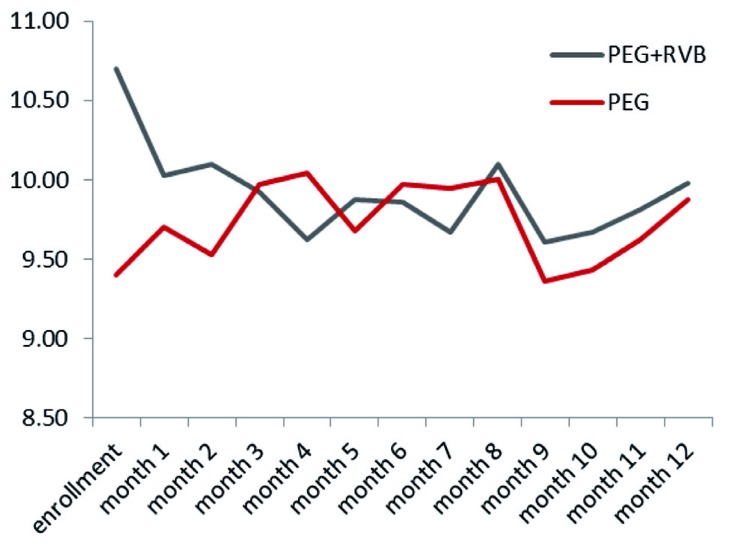
Pattern of Hemoglobin Change During Treatment in Both Treatment Arms

Follow up of patients from enrollment to 24 weeks after treatment cessation is shown in Figure 4. The peginterferon alfa-2a dose was reduced in 19 % of group (A) and 18 % of group (B) patients. Besides, the ribavirin dose fluctuated in group (A) patients according to their hemoglobin level. Compliance with treatment was similar in both groups, so were discontinuation rates (24 % in group (A) and 26 % in group (B). Adverse events were reported in 85 % of all studied patients [86 % in group (A) and 83 % in group (B)], and most of the events were considered to be related to treatment. Table 2 shows adverse events and laboratory abnormalities in detail according to the treatment regimen. The incidence was similar in both treatment groups. Four patients died because of sepsis, two patients died of diabetic ketoacidosis and two deaths occurred in patients with heart failure. It should be mentioned that diabetic ketoacidosis was attributed to lack of compliance with insulin use in both subjects. Discontinuation of treatment due to adverse events occurred in 4 % of both treatment arms (Figure 4). In patients who received combination therapy, transfusion intervals were almost halved to maintain hemoglobin above 10 g/dL. Consequently, hemoglobin level was remained constant during combination therapy (Figure 5). Regarding transfusion rate during therapy, patients who received ribavirin had a mean transfusion number of 54 packed cell while those who received monotherapy had 33 packed cells as mean transfusion. The rate of hemoglobin decline to below 8g/dL was comparable between two groups (43 % in ribavirin vs. 37 % in non-ribavirin group). The factors that influenced the number of transfusions were receiving ribavirin, duration of therapy (48 vs. 24 weeks and dropouts), history of splenectomy and inherent severity of thalassemia. Applying the generalized linear model of repeated measure showed that dynamics of hemoglobin during treatment in both treatment arms was not different significantly (P = 0.3) despite the higher transfusion rate in combination therapy group.

**Table 2:** Adverse Events in The Study Patients

	**Group A, No. (%)**	**Group B, No. (%)**
Death	3 (1.5)	5 (6)
Non-response	21 (10)	5 (6)
Dose Reduction	39 (19)	15 (18)
Neutropenia	33 (16)	11 (13)
Thrombocytopenia	6 (3)	4 (5)
Headache	46 (23)	20 (25)
Lethargy	41 (21)	11 (12)
Dizziness	23 (12)	8 (10)
Insomnia	34 (17)	12 (15)
Irritability	67 (34)	23(28)
Depression	15 (7)	10 (12)
Fatigue	60 (30)	19 (23)
Weight loss	12 (6)	3 (3.7)
Flue like syndrome	11 (5)	5 (6)
Myalgia	65 (33)	26 (32)
Arthralgia	59 (30)	23 (28)
Nausea	11 (5)	4 (5)
Diarrhea	13 (6)	4 (5)
Cough	19 (9)	9 (11)
Alopecia	85 (43)	33 (41)
Dry skin	26 (13)	21 (26)
Pruritus	19 (9)	3 (4)
Rash	5 (2)	3 (4)
Chills	23 (12)	13 (16)
Fever	48 (24)	23 (28)

### 4.4. Treatment Outcome in Children

From 30 patients aged between 11 and 18 years, 22 patients received oral ribavirin. Mean ALT, serum HCV-RNA and serum ferritin was 100 ± 10 IU/L, 650,000 ± 120,000 IU/mL, and 2383 ± 330 ng/mL respectively. Furthermore, half of children had genotype 1 infection in both groups and their ALT, HCV-RNA and serum ferritin levels were also similar. In respect of liver histology, 18 out of 30 children had severe liver fibrosis (HAI ≥ 3), and eight children had severe liver iron deposition (Pearl’s score 4). Regarding virological response, 14 out of 22 (64 %) children that received oral ribavirin attained SVR, while SVR was observed in six out of eight (75 %) children who received monotherapy. Withdrawal causes were as follows: four children in ribavirin group were prematurely withdrawn due to non-response, two because of laboratory abnormalities, and one because of severe depression. Among subjects in peginterferon monotherapy, one subject due to severe depression and one for familial reasons were withdrawn from the treatment. Twenty one (70 %) children experienced at least one clinical adverse event, while the most prevalent adverse events were flue like syndrome (70 %), arthralgia (17 %), and myalgia (20 %).

## 5. Discussion

This large prospective study showed that low dose ribavirin in chronically HCV infected thalassemia patients is safe, tolerable and effective. Our primary efficacy analysis showed that SVR rate was significantly higher in group (A) patients who received combination therapy with low dose ribavirin in comparison with patients of group (B) who received monotherapy. Furthermore, multiple logistic-regression analysis with adjustment for baseline characteristics revealed that low dose ribavirin was even a stronger independent predictor of a SVR than what had been shown in our primary analysis. In addition to ribavi-rin combination therapy, female gender, HCV type non-1 and age < 24 years were predictors of SVR rate in the total studied population. These findings are consistent with the results of previous studies [28][29][30]. Although in non-thalassemia patients, higher liver and serum iron content is determined to undermine virological response to anti-HCV therapy, no study except one case report has reported this among thalassemia patients [15][16][30][31][32][33]. Serum ferritin and liver pearl score did not reach a significance level for predicting SVR among total studied population in the present investigation. nonetheless, our subgroup analysis showed that patients with serum ferritin below 2006 ng/mL responded to ribavirin significantly higher than those with a lower level of serum ferritin. Analysis of liver pearl score did not display the same result. We believe that this is due to the fact that pearl score is not a sensitive measure of evaluating liver iron. According to our data, strict iron chelation to decrease serum and liver iron content before and during ribavirin therapy of thalassemia patients can strongly be advised. other factors related to treatment such as necessity of peginterferon dose reduction, discontinuation of treatment and compliance with therapy was rather similar in both treatment arms. More patients discontinued the treatments owing to an insufficient therapeutic response in group (A) rather than group (B). The higher rate of previous treatment failure in group (A), even with ribavirin, could be a possible reason for this finding. on the other hand, the rate of treatment discontinuation because of safety concerns and death was almost the same among both studied groups. Furthermore, the heterogeneous patient population with regard to being treatment naive versus experienced, and the absence of randomization might make it difficult to assess the efficacy of ribavirin, especially considering the relatively small difference of SVR rates between RBV and non-RBV groups. However, our multivariate analysis showed that the effect of ribavirin became significantly higher when we adjusted the comparison according to other baseline characteristics including previous treatment (OR = 2.2 after adjustment vs. 1.65 before adjustment). This finding displays a statistical advantage of RBV regardless of being treatment naive and experienced. As mentioned before, during treatment, 4 patients who had previous history of splenectomy died due to septicemia, two subjects died of diabetic ketoacidosis and two patients died because of heart failure. Indeed, our safety data suggest that thalassemia patients with significant concurrent life threatening comorbidities such as heart failure and insulin dependent diabetes mellitus, in addition to a history of splenectomy that makes the patients prone to certain infectious agents, may not be good candidates for anti-HCV therapy. The decision on treatment and monitoring of thalassemia major patients should be well individualized according to other conditions that accompany patients’ underlying hemoglobinopathy. Moreover, by close observation and frequent transfusion, the rate of severe hemoglobin drop can be minimized. According to the world medical association declaration of Helsinki regarding ethical principles for medical research involving human subjects, considering contraindication of ribavirin in thalassemia major patients, we did not carry out randomization or blindness. nevertheless, the final decision on therapeutic regimen was made by patients, after being well informed about different aspects of possible efficacy of ribavirin and its probable side effects. As presented in Table 1 most of major predictors of virological response including HCV RNA level, sex, weight, BMI, HCV type, age and liver histological findings appeared to be similar in both groups [29][34][35][36][37]. However, someone can argue that lack of randomization might underestimate the efficacy of peginterferon monotherapy or overestimate the safety of ribavirin. The reason is that patients with more severe thalassemia or more co-morbidity might have denied ribavirin treatment. This is unlikely in our opinion because comparison of baseline serum ferritin level shows that those who accepted ribavirin therapy had a significantly higher serum ferritin level and subsequently a more severe thalassemia than those who declined to receive ribavirin [38][39]. Moreover, our clinical records do not show that two groups of patients had a significant distinctive frequency of co-morbidities such as cardiovascular or endocrine diseases [29 (15 %) patients in monotherapy group and 19 (23 %) in combination therapy group]. In order to find out which groups of thalassemia patients derive the most benefits from ribavirin, a subgroup analysis was conducted. This analysis indicated that the major impact of low dose ribavirin was in thalassemia major patients with older than 24 years of age, a low serum ferritin (< 2006 ng/mL), a previous treatment failure, an elevated ALT and liver fibrosis of 0-4 HAI, history of splenectomy and viral load ≤ 600,000 IU/mL and HCV genotype 1. This later observation has been reported by others as well [20]. our subgroup analysis may indicate that therapy with low dose ribavirin should be considered as the treatment of choice in certain subgroups of thalassemia major patients but not all patients. 30 thalassemia children with HCV infection participated in our study. The total rate of SVR was 66 % among them and was significantly higher than adults’ SVR rate (in aggregation 45 %). In contrast to adults, our data of 22 children who were treated with ribavirin showed that ribavirin did not further increase SVR rate of peginterferon monotherapy (64 vs. 75 % of SVR) in patients less than 18 years of age. Furthermore, none of well-known factors that can influence virological response to anti-HCV treatment could predict the treatment outcome in children. In non-thalassemia children, genotype 2/3 infection had a higher rate of SVR than difficult to treat genotypes. However, we could not verify this finding in thalassemia major children [40][41]. In addition to virological outcome, our safety data revealed that peginterferon monotherapy or its combination therapy with ribavirin in children with thalassemia major is safe and well tolerated. According to the present study, adult thalassemia patients with HCV infection can be treated successfully with low dose ribavirin. Hence, we strongly advise combination therapy in thalassemia patients with aforementioned clinical characteristics. However, ribavirin does not seem to be beneficial in thalassemia patients below 18 years of age.
